# mTOR Complexes as a Nutrient Sensor for Driving Cancer Progression

**DOI:** 10.3390/ijms19103267

**Published:** 2018-10-21

**Authors:** Mio Harachi, Kenta Masui, Yukinori Okamura, Ryota Tsukui, Paul S. Mischel, Noriyuki Shibata

**Affiliations:** 1 Department of Pathology, Division of Pathological Neuroscience, Tokyo Women’s Medical University, Tokyo 162-8666, Japan; harachi.mio@twmu.ac.jp (M.H.); y.okamura.vtol.osprey@gmail.com (Y.O.); tsukuryo0525@gmail.com (R.T.); shibatan@twmu.ac.jp (N.S.); 2 Ludwig Institute for Cancer Research, University of California San Diego, La Jolla, CA 92093, USA; pmischel@ucsd.edu

**Keywords:** mTOR complex, metabolic reprogramming, cancer, microenvironment, nutrient sensor

## Abstract

Recent advancement in the field of molecular cancer research has clearly revealed that abnormality of oncogenes or tumor suppressor genes causes tumor progression thorough the promotion of intracellular metabolism. Metabolic reprogramming is one of the strategies for cancer cells to ensure their survival by enabling cancer cells to obtain the macromolecular precursors and energy needed for the rapid growth. However, an orchestration of appropriate metabolic reactions for the cancer cell survival requires the precise mechanism to sense and harness the nutrient in the microenvironment. Mammalian/mechanistic target of rapamycin (mTOR) complexes are known downstream effectors of many cancer-causing mutations, which are thought to regulate cancer cell survival and growth. Recent studies demonstrate the intriguing role of mTOR to achieve the feat through metabolic reprogramming in cancer. Importantly, not only mTORC1, a well-known regulator of metabolism both in normal and cancer cell, but mTORC2, an essential partner of mTORC1 downstream of growth factor receptor signaling, controls cooperatively specific metabolism, which nominates them as an essential regulator of cancer metabolism as well as a promising candidate to garner and convey the nutrient information from the surrounding environment. In this article, we depict the recent findings on the role of mTOR complexes in cancer as a master regulator of cancer metabolism and a potential sensor of nutrients, especially focusing on glucose and amino acid sensing in cancer. Novel and detailed molecular mechanisms that amino acids activate mTOR complexes signaling have been identified. We would also like to mention the intricate crosstalk between glucose and amino acid metabolism that ensures the survival of cancer cells, but at the same time it could be exploitable for the novel intervention to target the metabolic vulnerabilities of cancer cells.

## 1. Introduction

Proliferating cells require not only adenosine triphosphate (ATP) as an essential energy source, but also intracellular building blocks including nucleotides, fatty acids, and proteins, and a reprogrammed metabolism could serve to support the synthesis of macromolecules [1,2]. The Warburg effect is a hallmark phenomenon of cancer metabolism and relies on aerobic glycolysis to generate the energy needed for an array of cellular processes in contrast to normal differentiated cells on mitochondrial oxidative phosphorylation [3,4]. In other words, cancer cells are heavily dependent and addicted to glucose metabolism for their survival. Amino acids are another major determinant to support cancer cell proliferation. For example, cancer cells take up glutamine to survive or proliferate by promoting the production of nucleotides, fatty acids, and proteins [5,6,7], an anaplerotic process that replenishes a metabolic cycle.

Cancer cells take up a large amount of amino acids and glucose from the extracellular environment as a carbon and nitrogen source for protein and nucleotide synthesis [1,8]. In the process of tumor initiation and progression, cancer cells are exposed to harsh conditions such as hypoxia or nutrient depletion in the tumor microenvironment. To survive in this severe environment, cancer cells must sense and respond to the status of nutrient availability in the extracellular environment to coordinately regulate the gene expression for sustaining the cell proliferation as well as everting the various stress that halts the cell proliferation and induce cell death [9,10,11]. Thus, unveiling the mechanisms how cancer cells gather the information on environmental nutrient and facilitate their survival would shed new light on the molecular pathogenesis of cancer progression, which could be harnessed to identify the unrecognized addiction and vulnerability.

Here, we focus on mammalian/mechanistic target of rapamycin (mTOR) complexes, essential regulator of cell proliferation and metabolism, as a potential key player to play a role in sensing nutrients to drive the intracellular tumor-promoting signaling cascade through metabolic reprogramming and epigenetic shift, and a key node which should be therapeutically targeted as a new mode of treatment to interfere with cancer cell metabolism.

## 2. Metabolic Reprogramming as an Essential Hallmark in Cancer

The hallmarks of cancer are composed of six biological capabilities, which are acquired during the multistep evolution of human neoplasms [12]. The complexities of neoplastic disease are well explained by fundamental principles of the cancer hallmarks. Metabolic reprogramming is an emerging core hallmark of cancer [12], and similar alterations are also observed in rapidly proliferating cells such as immune cells under patho-physiological conditions [13]. Various intrinsic and extrinsic molecular signaling shifts the intracellular metabolism to support the demands of rapidly proliferating cells, including ATP generation to maintain energy, biosynthesis of macromolecules, and maintenance of reduction-oxidation (redox) reaction. The central hallmark of this reprogramming lies in the phenomenon that cancer cells undergo glycolysis even in the presence of sufficient oxygen, termed “the Warburg effect,” and there has been much interest in examining and comprehending the pathways that regulate the survival advantages conferred by this aerobic glycolysis [3]. Over the past decade, however, far more complex aspects of cancer metabolism have emerged, and the Warburg effect alone cannot well explain all the metabolic changes required for rapid cell growth, including aerobic glycolysis, glutaminolysis, altered lipid metabolism, de novo nucleic acid synthesis, and reactive oxygen species (ROS) management. For instance, as for ROS metabolism, nutrient deficiency, glucose deprivation and hypoxia induce ATP reduction and ROS overproduction, which could promote metabolic reprogramming in cancer cells [14]. Of interest, activated mTORC1 increased the level of ROS [15], suggesting that mTOR complex induces metabolic reprogramming via ROS production in response to unpreferable environments for cancer cells. On the contrary, mTORC1 regulates superoxide dismutase 1 (SOD1) activity through reversible phosphorylation in response to nutrients, which moderates ROS level and prevents oxidative DNA damage [16], and mTORC2 regulates the production of reduced form of nicotinamide adenine dinucleotide phosphate (NADPH) and glutathione (GSH), which could counteract the overproduction of ROS [17]. Further, epigenetic landscape including a shift in DNA and histone modifications are shaped and modulated by intermediary metabolites produced via metabolic reprogramming [13]. Therefore, the dynamic plasticity of metabolic reprogramming and epigenetic shift can converge to confer the survival advantage to cancer cells, but these alterations also render cancer cells vulnerable to interference with the metabolic and epigenetic network. Deciphering the molecular mechanism of the metabolic and epigenetic regulations in cancer could pave the way for therapeutic intervention, and the recent emerging evidences have revealed the essential regulatory role of mTOR complexes in metabolic reprogramming, the responsibility to microenvironments, and the subsequent epigenetic changes, which can result in cell survival in harsh metabolic conditions and provide therapeutic opportunities in cancer.

## 3. mTORC1 and mTORC2-Irreplaceable Partners in Cancer Metabolic Reprogramming

Genetic mutations to constitutively activate phosphoinositide 3-kinase (PI3K)-Akt-mTOR signaling are reported to reprogram cellular metabolism and tumorigenesis, including receptor tyrosine kinase (RTK) amplification and mutations, phosphatidylinositol 4,5-bisphosphate 3-kinase catalytic subunit alpha isoform (PIK3CA) mutation and phosphatase and tensin homolog deleted from chromosome 10 (PTEN) loss [18]. Among them, as a serine/threonine protein kinase essential for the cellular function, two distinct multi-protein complexes of mTOR associate the signaling from growth factor receptor with cell growth, proliferation, and survival. mTOR complex 1 (mTORC1), an established druggable target against cancer, phosphorylates and controls its substrates p70 ribosomal protein S6 kinase 1 (S6K1) and eukaryotic translation initiation factor 4E-binding protein 1 (4E-BP1) to promote protein translation as well as anabolic metabolism downstream of growth factor receptor-activated PI3K-Akt signaling [19,20]. The important role of mTOR complex 2 (mTORC2) has been gradually unraveled, especially in the field of metabolic homeostasis and cancer biology. mTORC2 has been considered to be responsive to growth factor signaling and the interaction of ribosome, and to function mainly through activating Akt by phosphorylating it on serine 473 (Ser473) [21,22]. It can also phosphorylate other AGC subfamily kinases including serum and glucocorticoid-inducible kinase 1 (SGK1) and protein kinase C (PKC). Recent studies demonstrated that mTORC2 regulates tumor progression, chemotherapy resistance, and genome DNA stability in cancer cells, playing an unrecognized, essential role in cancer biology [23,24]. Of note, these effects appear to be independent from canonical Akt-mediated signaling [25], indicating the importance of mTORC2 itself in cancer biology.

The structures of mTORC1 and mTORC2 are characterized by sharing some components: they share the catalytic mTOR subunit, as well as mammalian lethal with sec-13 (mLST8, also known as GβL) [26,27], the DEP domain containing mTOR-interacting protein (DEPTOR) [28], and the Tti1/Tel2 complex [29]. In contrast, regulatory-associated protein of mammalian target of rapamycin (raptor) [30,31] and proline-rich Akt substrate 40kDa (PRAS40) [32,33,34,35] are specific to mTORC1, while rapamycin-insensitive companion of mTOR (rictor) [26,36], mammalian stress-activated map kinase-interacting protein 1 (mSin1) [37,38], and protein observed with rictor 1 and 2 (protor1/2) [33,39,40] are specific components of mTORC2.

**Figure 1:**
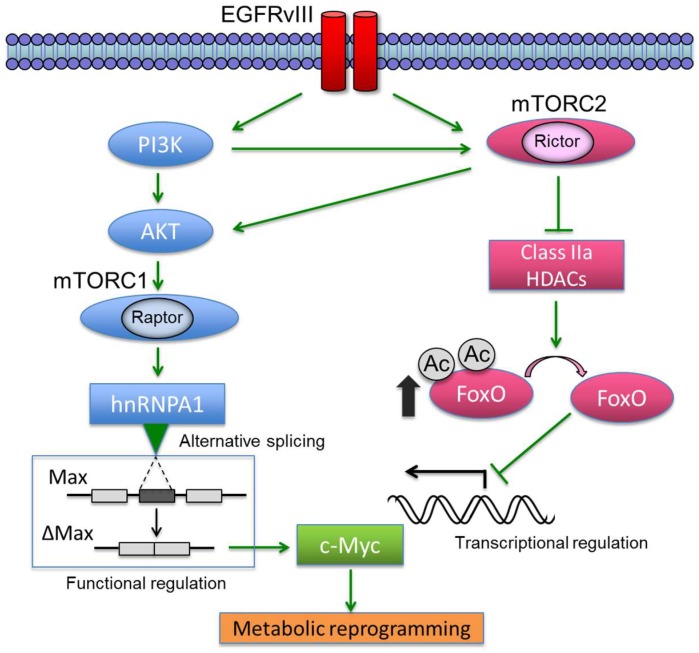
EGFRvIII controls c-Myc through two interlacing and synergistic mechanisms. EGFRvIII-mTORC1 signaling promotes glycolytic metabolism by activating hnRNPA1-dependent alternative splicing of a Myc-binding partner Delta Max, thereby functionally augmenting the oncogenic activity of c-Myc. Alternatively, EGFR-mTORC2 signaling controls c-Myc transcription, translation and protein level through FoxO acetylation, resulting in the enhancement of metabolic reprogramming. These findings point to the central role of c-Myc in regulating EGFRvIII-activated glycolytic metabolism. EGFRvIII: epidermal growth factor receptor variant III; PI3K: phosphoinositide 3-kinase; mTORC1/2: mammalian/mechanistic target of rapamycin complex 1/2; HDAC: histone deacetylase; hnRNPA1: heterogeneous nuclear ribonucleoprotein A1; Max: myc-associated factor X; FoxO: forkhead box O; Ac: acetyl-group.

Oncogenes and tumor suppressors are a key determinant in controlling cancer metabolism [41,42,43]. In various types of cancers, growth factor receptor signaling converges to an oncogenic transcription factor c-Myc, which promotes cell proliferation via metabolic reprogramming to connect nutrient uptake with intracellular biomass accumulation [44,45,46]. We recently identified that cancer cell metabolism was promoted through c-Myc which could be activated cooperatively by both mTORC1 and mTORC2 [47]. In glioblastoma (GBM, a malignant glial/astrocytic tumor) cells with activating epidermal growth factor receptor (EGFR) mutations, mTORC1 upregulates the heterogeneous nuclear ribonucleoprotein A1 (hnRNPA1) splicing factor, promoting the alternative splicing of Myc-associated factor X (Max) to generate Delta Max, thereby functionally augmenting Myc-dependent glycolytic metabolism and tumor cell proliferation [48]. mTORC2, on the other hand, increases the transcription of c-Myc through inhibitory phosphorylation of class IIa histone deacetylases (HDACs), resulting in inactivation of forkhead box O (FoxO) transcription factors through post-translational acetylation [47]. Therefore, growth factor receptor-PI3K signaling requires the synergistic action of mTORC1 and mTORC2 for c-Myc-dependent metabolic reprogramming by controlling both c-Myc transcription and its functional activity (Figure 1). Considering the essential and coupling roles of two mTOR complexes in reprogramming cancer cell metabolism, the next critical questions would be raised how mTOR complexes could sense the information on the source of metabolic reactions (i.e., nutrients) and subsequently respond to the microenvironmental condition to favor their survival.

## 4. mTORC1 as a Sensor of Amino Acids in Cancer Cells

mTORC1 is an evolutionarily conserved multi-protein complex that coordinates a network of signaling cascades and functions as a key mediator of protein translation, gene transcription, and autophagy [49,50,51]. mTORC1 is activated by growth factors such as insulin, and nutrients such as amino acids, which eventually promote cell growth and proliferation by regulating anabolic and catabolic processes and by driving cell cycle progression through phosphorylating its substrates [52,53]. However, when amino acid supplies become restricted, the activity of mTORC1 is significantly suppressed, and mammalian cells employ homeostatic mechanisms to rapidly inhibit processes such as protein synthesis, which demands high levels of amino acids. Additionally, mTORC1 supplies amino acid resource through releasing the suppression of autophagy under a starved state [54]. Of interest, the amino acid sensing mechanism that non-cancer cells use via mTORC1 could be exploited by cancer cells as described in the following sections, and the future endeavor should be directed to examine if there is actually a difference between cancer and non-cancer cells for amino acid sensing through mTORC1.

**Figure 2:**
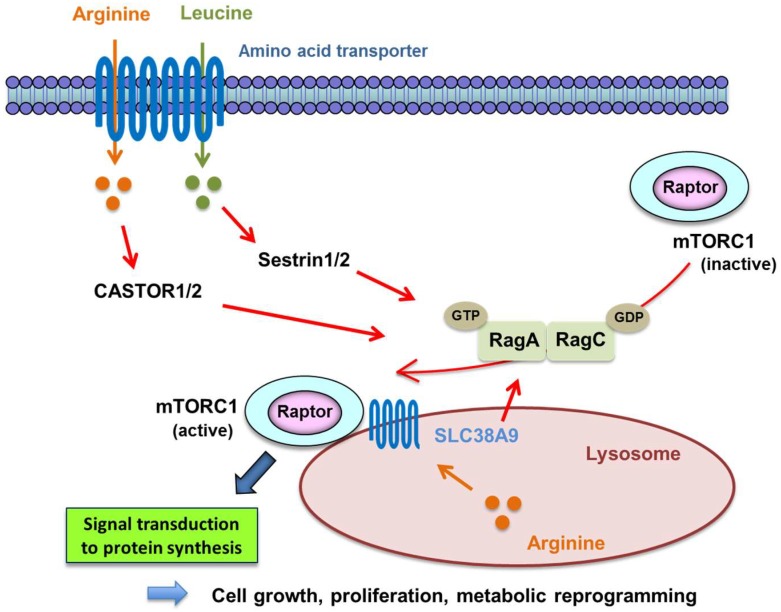
Mechanism of mTORC1 activation via Rag proteins by amino acids. mTORC1 is transferred to lysosome from cytosol by promoting heterodimerization of GTP-binding Rag proteins, which work as mediators of amino acid signaling to mTORC1. It is then activated by binding to GTP-bound Rheb on lysosome. Extracellular arginine and leucine activate RagA-RagC heterodimer, GTP-binding RagA, and GDP-binding RagC via amino acid transporter CASTOR1/2 and Sestrin1/2 to transfer mTORC1 to lysosome. Lysosomal arginine also activates Rag heterodimer via lysosomal amino acid transporter SLC38A9.

Leucine, one of the essential amino acids in human cells, mainly induces the recruitment of mTORC1 to the lysosomal membrane and its subsequent activation; that is, mTORC1 is activated in response to the level of leucine [55]. Leucine is also a signaling molecule that directly regulates animal physiology, including satiety [56], insulin secretion [57], and skeletal muscle anabolism [58,59]. Signal transduction through mTORC1, which is involved in cell growth through enhanced protein translation, is activated by extracellular leucine through Sestrin1/2, a GATOR2-interacting protein that inhibits mTORC1 signaling [55,60] (Figure 2). In addition to sensing leucine for its activation, CASTOR proteins were identified as a putative arginine sensor for the mTORC1 pathway, which is activated by extracellular arginine and interacts with GATOR2 and activate mTORC1 via promotion of the hetero-dimerization of GTP-RagA and GDP-RagC [61] (Figure 2). 

Recent studies also demonstrate the intriguing interaction of amino acid metabolism and mTORC1 signaling through the ubiquitin signaling systems, displaying that, in response to amino acids, the KLHL22 E3 ubiquitin ligase promotes K48-linked polyubiquitination, enabling mTORC1 signaling to promote tumorigenesis and aging [62]. Another interesting example is SAMTOR, which was reported to inhibit mTORC1 signaling by interacting with GATOR1, the GTPase activating protein (GAP) for RagA/B [63]. Notably, the methyl donor S-adenosylmethionine (SAM) disrupts the SAMTOR-GATOR1 complex, and methionine-induced activation of mTORC1 requires the SAM binding capacity of SAMTOR, indicating that mTORC1 is involved in methionine and one-carbon metabolism, which potentially control the epigenetic (methylation) shift in cancer. mTORC1 could thus respond to a range of amino acids and relevant metabolites, the mechanism of which could be involved in multiple human disease conditions including cancer. Importantly, the insufficiency for new protein synthesis is actively monitored by both prokaryotic and eukaryotic cells, and these amino acid sensing mechanisms described here might be an Achilles heel in cancer, which could be exploitable for the novel therapeutic strategies against cancer [64].

## 5. mTORC2 at the Intersection of Glucose and Amino Acid Metabolism

**Figure 3:**
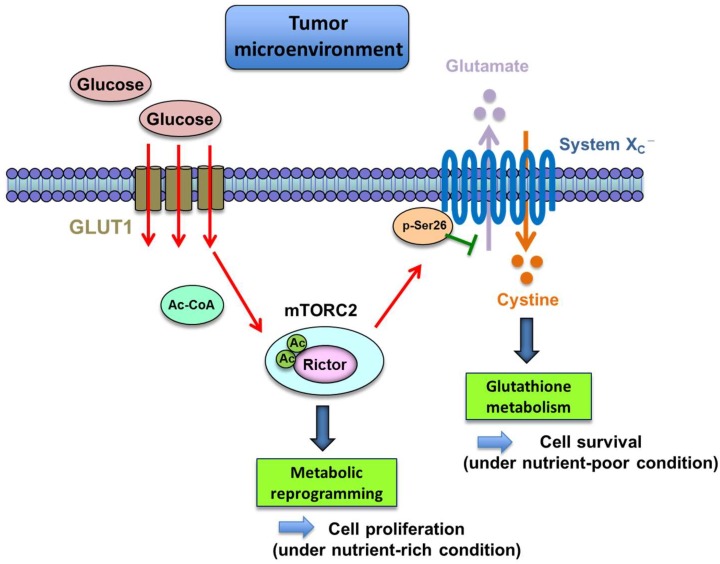
The function of mTORC2 as a sensor of glucose and amino acid. mTORC2 is activated by glucose through acetylation of Rictor, playing a role as a sensor of glucose. Phosphorylation of Ser26 of xCT by mTORC2 represses its function as glutamate-cystine anti-transporter. Under nutrient (glucose) poor conditions, lower mTORC2 signaling could tilt the balance from proliferation to survival by favoring glutamate efflux, cystine uptake, and glutathione synthesis to protect tumor cells from cellular stress.

Cancer cells convert the majority of glucose into lactate even under ample oxygen (the Warburg effect), the products of which could be used as carbon-containing precursors for the macromolecule production by rapidly proliferating cells. This intriguing phenomenon necessitates the presence of “glucose sensor” in cancer cells to precisely catch the information on the glucose in the microenvironment for appropriately responding to the environment and ensuring their survival. In recent years, several fascinating reports have been published, referring to the relationship between mTORC2, metabolic reprogramming, and nutrient sensing in cancer cells. mTORC2 is activated on the high-glucose extracellular condition via acetylation of Rictor, the main component of mTORC2 in human GBM cells [65] (Figure 3). This is metabolically mediated by the increased production of acetyl-coenzyme A (acetyl-CoA), a well-known donor of the acetyl group to the protein [66]. The findings suggest the possibility that mTORC2 could work as a potential glucose sensor in cancer cells.

Unlike mTORC1, which works as an amino acid sensor, the role of mTORC2 as a sensor of amino acid has yet to be clarified. However, using an unbiased proteomic screen, our recent work unraveled that mTORC2 could suppress the activity of the cystine-glutamate antiporter, system Xc transporter-related protein (xCT) via inhibitory phosphorylation of serine 26 of xCT’s N terminus cytosolic domain [67] (Figure 3). These results identify an unanticipated mechanism regulating amino acid metabolism in cancer, indicating that genetic mutations and aberrant signal transduction in cancer cells could reprogram amino acid metabolism. Additionally, this novel system would implicate the new role of mTORC2 as a potential amino acid sensor. Glutamine uptake, promoted by aberrant growth factor receptor and c-Myc signaling, is important for tumor cell proliferation, since it is subsequently converted to glutamate essential for tricarboxylic acid (TCA) cycle anaplerosis to provide a carbon source for proliferating cancer cells [65,68]. Thus, when the levels of exogenous nutrient are sufficiently high to support cancer cell proliferation, it would be of disadvantage for cancer cells to secrete glutamate, which is necessary for their anaplerotic reactions. Recently identified mechanisms here enable glutamine-derived glutamate to be utilized primarily for tumor cell proliferation when nutrients are rich in the tumor microenvironment. On the other hand, it would be an advantage for cancer cells to increase xCT-dependent cystine uptake in exchange for glutamate efflux, enabling tumor cells to neutralize the cellular oxidative stresses by synthesizing glutathione from xCT-derived cysteine, when extracellular nutrients are lacking. Therefore, the mechanism on amino acid metabolism described here makes it possible for cancer cells to respond and adapt to a dynamic shift in nutrient levels in the tumor microenvironment.

An unexpected implication for cancer cell metabolism comes from these recent studies regarding the sensing mechanism of glucose and amino acid by mTORC2. Exogenous nutrients including glucose and acetate have been reported to activate mTORC2 to phosphorylate its downstream substrates as aforementioned [69], and this mTORC2-dependent glucose sensing mechanism would raise the possibility that, when extracellular nutrients become scarce, lower mTORC2 signaling could tilt the balance of tumor cell status from cell proliferation to cell survival, at least partly by preferring glutamate efflux, cystine uptake, and glutathione synthesis in order to protect tumor cells from the oxidative cellular stresses, the mechanism of which is based on the mTORC2-dependent regulation of xCT systems (Figure 3). The findings provide the challenging and promising ideas on the previously unrecognized interaction between glucose and amino acid metabolism through mTORC2 signaling, which enables cancer cells to promote their survival according to the level of nutrients in the microenvironment.

## 6. Epigenetic Modulation by mTOR-Dependent Metabolism in Cancer

Many enzymes that play important roles in epigenetic gene regulation utilize intermediary metabolites as co-substrates yielded by cellular metabolic reprogramming [70]. Indeed, epigenetic modifiers are sensitive to alterations in the levels of multiple intermediary metabolites, which can be regulated by PI3K/Akt/mTOR signaling.

Acetylation on the N-terminal lysine tail of histones leads to the neutralization of positively charged lysine with an open chromatin configuration facilitating transcription. On the other side of the coin, deacetylation of histones is associated with condensed chromatin and reduction of transcriptional activity. Acetyl-CoA is the substrate used to modify histone tails, and can be produced through a variety of metabolic pathways. Its primary generation sources are through the conversion of pyruvate from glycolysis and citrate from the TCA cycle. Intriguingly, some of the processes seem to be governed by the RTK/PI3K/Akt/mTOR pathway, thus possibly linking their metabolic processes to epigenetic status. Recent studies demonstrated that dynamic translocation of mitochondrial pyruvate dehydrogenase complex (PDC) to the nucleus provides a pathway for nuclear acetyl-CoA synthesis required for histone acetylation and epigenetic regulation [71]. Interestingly, the nuclear translocation of PDC seems to be facilitated by the stimulation of growth factor receptor signaling, and it may be mediated by mTOR pathway signaling. ATP citrate lyase (ACLY) is a key enzyme responsible for generating cytosolic acetyl-CoA and oxaloacetate. Akt enhances the phosphorylation and activation of ACLY, and ACLY inhibition results in tumor growth arrest [72]. ACLY is also regulated by growth factor stimulation, which is required for histone acetylation and gene expression [73]. Thus, recent discoveries that class IIa HDACs (HDAC4, 5, 7, and 9) are involved in glucogenic metabolic processes [74] and are regulated by mTORC2 [47] suggest that mTORC2 may affect histone acetylation directly or indirectly through the regulation of acetyl-CoA producing as well as histone modifying enzymes. Intracellular acetyl-CoA also derives from β-oxidation of fatty acids. Recent work has highlighted the importance of fatty acid catabolism in cellular energy homeostasis, and it may also affect the epigenetics through the production of acetyl-CoA. In cancer cells, a gene expressed only in the brain, carnitine palmitoyltransferase 1C, was reported to promote fatty acid oxidation and cell survival, and confer rapamycin resistance, indicating that this gene may act in parallel to mTOR-enhanced glycolysis [75].

In addition to histone acetylation, histone methylation is also important in defining the epigenetic state of chromatin as well as the methylation of DNA itself. A methyl-donor SAM derived from methionine is utilized by methyltransferases; thus, its metabolism can profoundly affect the DNA and histone methylation status. Methionine adenosyltransferase (MAT) is an essential enzyme responsible for SAM biosynthesis, and the function and subclass switch of MAT can be associated with PI3K/Akt signaling, affecting global DNA methylation and cell survival in cancer [76]. Recently, HDAC4 (class IIa HDAC) is reported to play a central role for histone methylation in response to cardiac load, revealing a new relationship between HDACs and histone methylation [77], so mTORC2 might be involved in the regulation of global histone methylation through the inhibition of class IIa HDACs [47].

Somatic mutation of the NADP(+)-dependent enzyme isocitrate dehydrogenase (IDH) is frequently found in cancer, and is shown to acquire a neomorphic enzymatic activity that converts α-ketoglutarate (α-KG) to 2-hydroxyglutarate (2-HG) [78]. Oncometabolite 2-HG was shown to affect the epigenetic status through the inhibition of α-KG-dependent dioxygenase/DNA demethylase (TET2) for DNA methylation and the Jumonji-domain-containing protein 2A (JMJD2A/KDM4A) for histone methylation, eventually contributing to the genome-wide methylator phenotype (CIMP: CpG island methylator phenotype). 2-HG also activates the EGLN1 prolyl hydroxylase and increases the degradation of HIF. The PI3K/Akt/mTOR pathway is relevant to HIF regulation [13], which might further connect metabolism to epigenetics through the control of epigenetic enzymes by HIF and counter-balance the IDH-mediated epigenetic changes. Intriguingly, recent reports demonstrated that DNA methylation landscape of cancer progression shows extensive heterogeneity in time and space [79,80], and further comprehension of these relationships will help our understanding of the mechanics of a variety of metabolic diseases including cancer.

## 7. Molecular Therapies Targeting mTOR-Dependent Signaling and Metabolism

**Figure 4:**
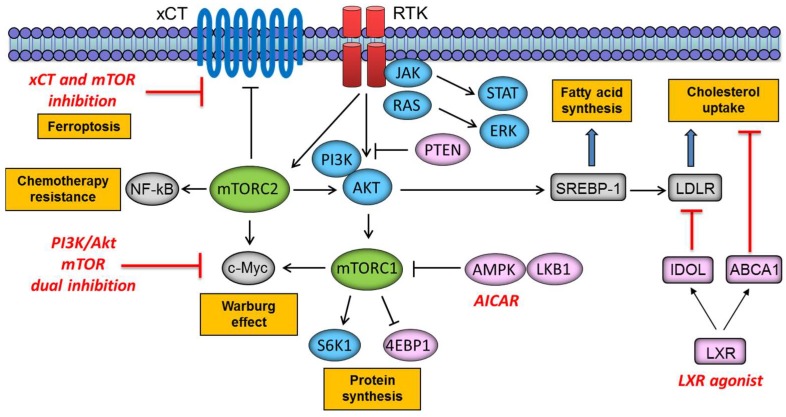
Potential molecular therapies targeting mTOR-dependent metabolic reprogramming in cancer cells. xCT: amino acid transport system Xc-; NF-κB: nuclear factor-kappa B; RTK: receptor tyrosine kinase; JAK: Janus kinase; STAT: signal transducers and activators of transcription; ERK: extracellular signal-regulated kinase; S6K1: ribosomal protein S6 kinase 1; 4EBP1: eukaryotic translation initiation factor 4E-binding protein 1; LKB1: liver kinase B1; AMPK: adenosine monophosphate-activated protein kinase; SREBP-1: sterol regulatory element-binding protein 1; LDLR: low density lipoprotein receptor; IDOL: inducible degrader of LDLR; ABCA1: ATP-binding cassette protein A1; LXR: liver X receptor.

Understanding the complex role of mTOR in regulating signal transduction is critical to developing more effective therapies to target metabolic reprogramming in cancer. Distinctly, rapamycin treatment (a macrolide antibiotic and immunosuppressive compound that inhibits mTORC1 signaling) leads to the release of Akt suppression (hence activation of mTORC2), due to the loss of negative feedback for attenuating PI3K signaling [81]. The PI3K pathway reactivation after rapamycin treatment indicates that dual PI3K/mTOR inhibitors function by preventing PI3K signaling reactivation and more effectively target mTORC2 (and mTORC1) signaling [82]. Our study further sheds light on the resistant mechanisms of GBM to targeted therapies, providing compelling rationale for the combined inhibition of PI3K/Akt and mTORC2 as a promising “combinatorial targeted therapy” for targeting cancer cell metabolism [83] (Figure 4). We recently demonstrated that EGFRvIII and loss of PTEN potently activate mTORC2, resulting in GBM cell growth and survival by activating NF-κB through SGK1. This study also identified a previously unsuspected role for mTORC2 in mediating chemotherapy resistance, and EGFRvIII-expressing GBMs are exquisitely resistant to cisplatin, temozolomide, and etoposide [24]. These results strongly suggest a critical role for drugs that target both mTORC1 and mTORC2, including in combination with chemotherapy.

mTOR also plays a critical role in integrating cellular metabolism with signal transduction. mTORC1 has emerged as a critical effector downstream of the tumor suppressor liver kinase B1 (LKB1). LKB1 is thought to suppress tumor formation by negatively regulating mTORC1 signaling through adenosine monophosphate (AMP)-activated protein kinase (AMPK). A study by our group demonstrated that the AMPK activator, 5-aminoimidazole-4-carboxamide-1-β-d-ribonucleoside (AICAR) effectively blocks the growth of EGFR-activated GBM primarily by inhibiting lipogenesis [84] (Figure 4). We also demonstrated that EGFR signaling promotes activation of the transcriptional regulator of fatty acid synthesis, sterol regulatory element-binding protein-1 (SREBP-1) [85]. Further investigation uncovered an EGFRvIII-activated, PI3K/SREBP-1-dependent tumor survival pathway through the low-density lipoprotein receptor (LDLR) [86]. Targeting LDLR with the liver X receptor (LXR) agonist caused an inducible degrader of LDLR (IDOL)-mediated LDLR degradation and increased expression of the ATP-binding cassette protein A1 (ABCA1) cholesterol efflux transporter, potently promoting tumor cell death in a GBM model (Figure 4). Further, GBM is remarkably dependent on cholesterol for survival, rendering these tumors sensitive to LXR agonist-dependent cell death, based on identifying and targeting tumor co-dependencies shaped both by aberrant EGFR-mTOR signaling and the brain’s unique biochemical environment [87]. Recent reports also demonstrate a novel link between mTOR complexes and lipid metabolism. mTORC2 stimulated sphingolipid and glycerophospholipid synthesis, and inhibition of fatty acid or sphingolipid synthesis prevented tumor development, indicating a causal effect in tumorigenesis as well as a novel therapeutic opportunity [88]. Further, in addition to the role of mTOR a sensor of amino acids and glucose, a recent study reveals that mTOR also senses the presence of lipids through production of phosphatidic acid, expanding its role as a metabolic sensor in the cell [89]. Additionally, the combination of an xCT inhibitor erastin with Torin1 (mTOR kinase inhibitor, which blocks both mTORC1 and mTORC2 activity) resulted in significant GBM cell death, while cell survival was not affected by either drug alone, indicating that increased xCT activity has a major contribution to glutathione synthesis and GBM cell survival upon pharmacological mTOR kinase inhibition [67] (Figure 4). Thus, understanding the regulation of cellular metabolism with mTOR signaling may pave the way for the development of more effective treatment strategies.

## 8. Unanswered Questions on mTOR-Dependent Metabolism in Cancer

There have traditionally been plenty of the epidemiological studies endeavoring to reveal the statistic link between cancer incidence and metabolic factors including obesity, diabetes mellitus, and Western-type life style and diet. However, the relationship was not usually solid, partly because most of the studies did not touch on the tumor genotype. Future studies will be necessary to plan the mechanistic types of studies that will untangle the interaction between nutrients, metabolism, and cancer biology on a genetic and molecular basis, which will eventually reveal the impact of nutrient and metabolism on tumor pathogenesis, aggressiveness, and response/resistance to treatment. Thus, mTOR-dependent metabolism should be evaluated in the specific context of genotype-defined and nutrient/environment-restricted conditions.

We have proposed the questions on mTOR-dependent metabolism in cancer, which should be tackled in the future for achieving the goal of developing novel therapeutic and preventive strategies against cancer with facilitated metabolism. In order to determine whether accelerated cellular metabolism is not only a consequence reprogrammed by oncogenic signaling but also potentially affected by a specific tumor microenvironment, it will be necessary to regulate diet and nutrient levels in genotyped human and mouse tumor models to assess the impact of metabolism on signal activation, tumor progression, and response/resistance to treatment. The critical point here is to recognize that the status of specific nutrients in the tumor microenvironment are not merely a consequence of the diet but rather established by the intricate interaction between diet uptake, de novo/salvage synthesis, and cellular utilization [90]. Thus, directly measuring the levels of specific nutrient and tracing their uptake and utilization in tumor tissue in human and genetically engineered mouse models will be needed. Importantly, mTOR complexes are one of the critical hubs and nodes integrating nutrient status and altered growth factor receptor signaling, but understanding how they interact with other nutrient sensing pathways will also be important.

In addition, to test a hypothesis that mTOR complexes are a key node to integrate growth factor receptor signaling with nutrient availability, influencing tumor growth and response to treatment, it will be necessary to study the role of mTOR and its modulation by nutrients in various cancer types. Furthermore, to test the hypothesis that glucose or amino acid-derived intermediates including acetyl-CoA and SAM directly contribute to tumor growth and drug resistance, genetic studies will be needed particularly to confirm the hypothesized importance of histone acetylation, histone methylation, and DNA methylation and to determine if persistent mTOR signaling is sufficient to maintain tumor growth and cause drug resistance through metabolic reprogramming and subsequent epigenetic shifts, for example, by examining the impact of elevated acetyl-CoA and SAM levels on enhancer activation and transcriptional reprogramming.

## 9. Conclusions and Future Perspectives

The intricate orchestration of responses that enable cancer cells to meet their demands in a completely cell-autonomous fashion defines the specificity of metabolic reprogramming in cancer. A promising and less toxic therapeutic strategy for patients with cancer will be achieved by the development of inhibitors that target cancer-specific signal transduction and metabolism. However, that goal is unlikely to be achieved until the impact of cancer-causing driver mutations on metabolic reprogramming and epigenetic regulation are deeply comprehended, including the flexible ways in which tumor cells appropriately sense the nutrient status in microenvironment and adapt to changing conditions, so as to coordinately sustain the constitutive activation of downstream effectors necessary for tumor cell proliferation and survival. We have herein summarized the recent literature, clearly pointing out an unanticipated important role for both mTOR complexes in sensing essential nutrients including glucose and amino acids via cancer metabolic reprogramming, where they integrate aberrant signaling activities into biochemical reactions and potential transcriptional regulations that drive tumor progression. We have also highlighted an emerging role for mTORC2 in linking aerobic glycolytic metabolism with amino acid metabolism, which could potentially be a key mechanism to exquisitely tilt the balance between dichotomic cellular events essential for tumor cell survival including cell proliferation and cell protection from oxidative stress. Future endeavors should be directed to understand how driver mutations in cancer rewire intracellular signaling cascades to translate biochemical metabolic reactions into global epigenetic ensembles [17]. Cooperative, multidisciplinary, and translational approaches would be necessary to yield critical insights into the etiology and pathogenesis of cancer, shed new light on how tumor cells resist molecularly targeted therapies, and possibly pave the way for the development of more effective signaling-dependent metabolism-targeted treatments against cancer to achieve the goal of “precision medicine.”
